# Opposite Role of Kindlin-1 and Kindlin-2 in Lung Cancers

**DOI:** 10.1371/journal.pone.0050313

**Published:** 2012-11-29

**Authors:** Jun Zhan, Xiang Zhu, Yongqing Guo, Yunling Wang, Yuxiang Wang, Guangliang Qiang, Miaomiao Niu, Jinxia Hu, Juan Du, Zhilun Li, Jia Cui, Bo Ma, Weigang Fang, Hongquan Zhang

**Affiliations:** 1 Key Laboratory of Carcinogenesis and Translational Research, Ministry of Education of Beijing, Beijing, People's Republic of China; 2 Laboratory of Molecular Cell Biology and Tumor Biology, Department of Anatomy, Histology and Embryology, Peking University Health Science Center, Beijing, People's Republic of China; 3 Department of Pathology, Peking University Health Science Center, Beijing, People's Republic of China; 4 Institute of Cardiovascular Research, Peking University Health Science Center, Beijing, People's Republic of China; 5 Department of Thoracic Surgery, Sino-Japan Friendship Hospital, Beijing, People's Republic of China; 6 Unit for Clinical Molecular Biology, Department of Biosciences and Nutrition, Karolinska Institutet, Huddinge, Sweden; Cincinnati Children's Hospital Medical Center, United States of America

## Abstract

Lung cancer is highly heterogenous and is composed of various subtypes that are in diverse differential stages. The newly identified integrin-interacting proteins Kindlin-1 and Kindlin-2 are the activators of transmembrane receptor integrins that play important roles in cancer progression. In this report we present the expression profiles of Kindlin-1 and Kindlin-2 in lung cancers using patient specimens and established their correlation with lung cancer progression. We found that Kindlin-1 was expressed in epithelia-derived non-small-cell lung cancer, especially in squamous cell lung cancer but expressed at low levels in poorly differentiated large cell lung cancer. However, Kindlin-2 was highly expressed in large cell lung cancer. Both Kindlin-1 and Kindlin-2 were found not expressed or expressed at very low levels in neuroendocrine-derived small cell lung cancer. Importantly, the Kindlin-1 expression level was positively correlated with the differentiation of squamous cell lung cancer. Surprisingly, we found that the very homologous Kindlin family proteins, Kindlin-1 and Kindlin-2, displayed counteracting functional roles in lung cancer cells. Ectopic expression of Kindlin-1 in non-small-cell lung cancer cells inhibited *in vitro* cell migration and *in vivo* tumor growth, while Kindlin-2 promoted these functions. Mechanistically, Kindlin-1 prohibited epithelail to mesenchymal transition in non-small-cell lung cancer cells, while Kindlin-2 enhanced epithelail to mesenchymal transition in these cells. Taken together, we demonstrated that Kindlin-1 and Kindlin-2 differentially regulate lung cancer cell progression. Further, the expression levels of Kindlin-1 might be potentially used as a marker for lung cancer differentiation and targeting Kindlin-2 might block the invasive growth of large cell lung cancer.

## Introduction

Lung cancer is a complicated disease that can be histologically classified into small cell lung carcinoma (SCLC) which represents nearly 20% of lung cancer, and the major part is non-small -cell lung cancer (NSCLC) which represents more than 80% of lung cancer [1]. NSCLC encompass squamous cell carcinoma (SCC), adenocarcinoma (AC), and large cell carcinoma (LCC) [2]. Highly differentiated, moderate differentiated and poorly differentiated carcinomas can be seen in SCC and AC. Lung cancer is quite heterogeneous in tumor growth, invasion and metastasis as well as in the outcomes after treatment. Lung cancer in different differentiation status displayed discrepancy in malignancy, e.g., low-differentiated lung carcinomas are more malignant than high-differentiated cancers, which is fast growing and highly metastatic.

Fermitin family member 1 (FERMT1) encoding Kindlin-1 is a FERM (4.1-Ezrin-Radixin- Moesin)-containing protein that belongs to Kindlin family. Kindlins are integrin- interacting proteins that regulate integrin activation via interaction with the integrin β subunit cytoplasmic domain [3], [4]. Kindlin-2 encoded by FERMT2 was found to control a bidirectional signaling via integrin [5]. Kindlin was designated from Kindler Syndrome that was coined in 1954 featured by a combination of epidermal atrophy, widened capillaries and mottled cutaneous pigmentation [6]. Mutations of Kindlin-1 had been identified as the cause of Kindler Syndrome [7], [8]. Until recently Kindlin family member have been connected to the progression of tumors [9]. Kindlin-2 has been shown to be expressed in malignant mesothelioma and regulates adhesion and migration [10], and the expression level of Kindlin-2 is related to the sensitivity of prostate cancer cells to cisplatin-induced cell death [11]. Kindlin-2 expression was found correlated with tumor invasion, lymph node metastasis, and patient outcome in gastric cancer [12]. Kindlin-1 has been found overexpressed in 60% of lung cancer and 70% of colon cancer as examined by RNA expression levels out of ten patients [9]. Very recently, Sin et al demonstrated that Kindlin-1 plays a role in breast cancer growth and lung metastasis [13]. Besides expression in tumor cells, Kindlin-2 was also found to be highly expressed in the tumor stroma in bladder cancers [14]. However, while Kindlin-1 and Kindlin-2 are related to cancer progression, Kindlin-3 is contributory in the hematopoietic disorders [15]–[19]. Kindlin-2 expressed broadly in endothelial and vesicular smooth muscle [3], [20], and is an essential component of intercalated discs that is required for vertebrate cardiac structure and function [21]. However, so far little is known about the roles of Kindlin-1 and Kindlin-2 in lung cancer progression.

In this study we aim to answer firstly that if Kindlin-1 and Kindlin-2 play a role in lung cancer progression. Due to the high heterogenecity of lung cancer we also want to answer that if Kindlin-1 and Kindlin-2 differentially expressed in various types of lung cancer and functions distinctly in lung cancer cells. To this end, we have investigated the expression of Kindlin-1 and Kindlin-2 in a panel of lung cancer tissue samples and paid attention to compare the relative expression levels of Kindlin-1 and Kindlin-2 in the same patient. Intriguingly, we identified a reciprocal role of Kindlin-1 and Kindlin-2 in the regulation of tumor progression in both cells and animals. Taken together, our findings provide a new understanding of Kindlin-1 and Kindlin-2 in lung cancer cell progression and may help for future drug design towards lung cancer therapeutics.

## Materials and Methods

### Ethics

The Ethics Committee of Peking University Health Science Center has approved the current study for mouse experiments (Permit Number: LA2011-73). The Ethics Committee of Sino-Japan Friendship Hospital has approved the current study using lung cancer patient tumor tissues for research purposes (Permit Number: ZRLW-5).The procedures for handling mice and human materials were in accordance with the ethical standard of the Helsinki Declaration of 1975, and the revised in 1983.

### Expression vectors, cell culture and stable transfection cell lines

Kindlin-1 full-length cDNA was cloned from a human placenta cDNA library using primers: CAGAATCCATGCTGTCATCCACTGACTTTAC (forward primer) and GATCTAGATCAATCCTGACCGCCGGTCAA (reverse primer). PCR product was cloned into PCRII vector, and then further cloned into pCMV10-3×Flag vector (Sigma) using HindIII-EcoRI sites. U1752, H1299, A549 and U1810 cell lines were purchased from ATCC in the USA or Cell Collection Center of Peking Union Medical School in China and were cultured in RPMI1640 medium (Invitrogen) with 10% FBS and 50 µg/ml gentamycin. Cells were grown in 75 cm^2^ culture flasks or 60-mm dishes at 37°C in humidity with 5% (v/v) CO_2_. Media were changed every two days. For establishment of stable clones that expressed Kindlin-1 and Kindlin-2 separately, H1299 cells were transfected with Flag-Kindlin-1, Flag-Kindlin-2 and empty vector using Lipofectamine. Twenty-four h after transfection cells were passaged and G418 was added at final concentration of 1200 µg/ml. Single clones that expressed Kindlin-1 or Kindlin-2 were pooled together to avoid clonal effects. Pooled cells expressing Kindlin-1 or Kindlin-2 were used for functional studies as indicated in the text.

### Tumor tissues

Lung cancer tissue specimens were obtained from the Department of Thoracic Surgery Sino-Japan Friendship Hospital, Beijing China, and were sectioned at the Department of Pathology Health Science Center Peking University, Beijing China. Among the 140 tumor tissue sections that were used for immunohistochemical analyses, 65 are SCC, 43 are AC, 12 are LCC and 20 are SCLC (Table 1). All of these samples were subjected to Kindlin-1 and Kindlin-2 staining. In addition, 7 fresh lung cancer tissues with the paired tumor vicinity tissues include 3 SCC, 3 AC and 1 LCLC. These tissues were used to extract mRNA for qPCR detection of Kindlin-1 expression.

**Table 1:** Clinical-pathological data of lung cancer patients.

Feature			No. of Patients
			n = 140
Sex:	Male		104
	Female		36
Age:	30–39		1
	40–49		10
	50–59		33
	60–69		58
	70–79		35
	80-		3
Pathological diagnosis:			n = 140
Non- small cell lung carcinoma	Squamous cell lung carcinoma	Well differentiated	4
		Moderate differentiated	50
		Poorly differentiated	11
	Adenocarcinoma	Well differentiated	7
		Moderate differentiated	28
		Poorly differentiated	8
	Large cell carcinoma		12
Small cell lung carcinoma			20

### Western blot analysis

PBSTDS lysis buffer with the presence of protease cocktail inhibitor (Roche Diagnostics, GmbH) was used to extract total cell lysates. 80% of cell lysates was added to 20% of 5× SDS loading buffer, then resolved by 10% SDS-PAGE gel and blotted onto PVDF membranes (pore size 0.45 µm). The primary antibodies anti-Kindlin-1 mouse monoclonal antibody (1∶500 dilution; Clone 4A5.14, Milipore, USA), anti-Kindlin-2 mouse monoclonal antibody (1∶1000 dilution; Clone 3A3, Milipore, USA), anti-Vimentin rabbit monoclonal antibody (1∶ 2000 dilution; Epitomics, USA), anti-Ecadherin rabbit monoclonal antibody (1∶ 3000 dilution; Epitomics, USA), anti-N-caherin rabbit monoclonal antibody (1∶ 10000 dilution; Epitomics, USA), anti-Flag-monoclonal antibody (1∶10000 dilution; Clone M2, Sigma, USA) and anti-β-actin mouse monoclonal antibody (1∶1000 dilution; Clone C4, Santa Cruz, USA) were incubated with the membranes separately under rotation. After thorough washing, membranes were further incubated with corresponding secondary antibodies recognizing either rabbit or mouse Ig (Jackson Laboratories, USA). Finally, the bands were visualized by the enhanced chemoluminescence (Pierce).

### Quantitative PCR

Quantitative PCR (qPCR) assays were performed to detect the expression of Kindlin-1 mRNA in lung tumor tissues. In brief, the normal tissue and lung tumor total mRNAs were isolated by Trizol (Invitrogen, Carlsbad, CA, USA), and 2 µg of total RNA was reverse transcribed using M-MLV reverse transcriptase (Promega, CA, USA). Then PCR was performed using Taq PCR MasterMix (TIANGEN, China) with the settings as: 94°C 2 min; 94°C 30 sec, 60°C 30 sec, 72°C 30 sec, for 30 cycles; 72°C 5 min. The primer sequences were as follows: for human Kindlin-1, forward: TCATGTTGGAGGAGTGATGC, reverse: AAGCCAGCAATGCTTCTGTT; for actin, forward: CTGAGCGTGGCTACTCCTTC, reverse: GCCATCTCGTTCTCGAAGTC. PCR products were analyzed by 2.5% agarose gel electrophoresis in the presence of ethidium bromide for visualization. Quantitative PCR was also used to identify the changes of MMPs mRNA level induced by Kindlin-1 and Kindlin-2 in H1299 cell line. MMP-2 forward: AGCTCCCGGAAAAGATTGAT, reverse: GGTGCTGGCTGAGTAG- ATCC; MMP-7 forward: GTCTCGGAGGAGATGCTCAC; reverse: TACCCAAA-GAATGGCCAAGT; MMP-9 forward: TCTTCCCTGGAGACCTGAGA; reverse: ATTTCGACTCTCCACGCATC. Relative fold changes in qPCR were determined by the ΔΔCt method.

### Cell motility assays

Transwell chambers (Costar) with 8 µm pore size were used to perform the cell migration assay. First, Collagen type I were used to coat the lower surface of Transwell membranes for 1 h at room temperature. Then, H1299 cells stable transfected with Flag-BAP (bacterial alkaline phosphatase), Flag-Kindlin-1 and Flag-Kindlin-2 were seeded on the upper surface of the Transwell. After 6 h incubation in migration buffer (RPMI1640, 2 mM CaCl_2_, 1 mM MgCl_2_, 0.2 mM MnCl_2_, and 0.5% BSA) at 37°C in humidified 5% CO_2_, the Transwell membranes were fixed with 4% formaldehyde for 15 min and stained by crystal violet for 10 min. At last, 6 microscopic fields were randomly chosen for counting the migrated cells.

For cell wound healing experiment, 48 h after transfection monolayer cells were scratched using a standard 100 µl pipette tip. The wounded monolayers were washed twice to remove non-adherent cells with 0.1 M PBS (pH7.2). Twelve h later the wound was observed with a 20×objective (Nikon, Japan) and measured for the width of the cell wounds. Six microscopic fields for each dish were observed and photographed.

### Immunohistochemistry (IHC)

Lung tumor slides obtained from Sino-Japan Friendship Hospital were all formalin-fixed and paraffinembeded. Deparaffinization and hydration were performed and followed by abolishing endogenous peroxidase activity using 0.3% hydrogen peroxide for 30 min and microwave for antigen retrieval in 10 mM sodium citrate buffer (pH6.0) for 20 min. We used affinity-purified polyclonal anti-Kindlin-1 (PKU Animal Facility) antibody at 2 µg/ml and monoclonal anti-Kindlin-2 (Milipore, USA) at 2 µg/ml to perform these experiments. The primary antibody was used at 4°C overnight. Then PV9000 2-step plus Poly-HRP Anti-mouse/rabbit IgG Detection System (Zhong Shan Jin Qiao) was applied. The streptavidin-biotin- peroxidase method was used for detection and diaminobenzidine was applied for substrate (ChemMate Detection Kit, DAKO). Hematoxylin was used for counterstain. Negative controls were performed by omitting the use of primary antibody.

### Evaluation of immunohistochemistry

Two independent pathologists evaluated all immunostainings and a consensus justification based on discussion was recorded. The assessment was classified into 4 grades: no reactivity marked as 0, faint reactivity as 1+, moderate reactivity as 2+, and strong reactivity as 3+.

### *In vivo* xenograft tumor growth experiment

Balb/c nude mice were implanted subcutaneously into the flank with 1×10^6^ cells which are stably overexpressing Kindlin-1, Kindlin-2 and the control vector separately. Tumors were measured for their sizes in eighteen days and continue to record for the indicated time. Mice were sacrificed using euthanasia when tumor growth at 1 cm in diameter. Tumors were taken out and weighted.

### Statistical analysis

Statistical analyses for patient materials were performed with Kruskal-Wallis test and Statistical analyses for paired samples employed Student's t test. All statistical analyses were using SPSS version14.0. Results were considered statistically significant at the level of *P*<0.05.

## Results

### Kindlin-1 is expressed in normal lung tissue and different subtypes of lung cancer

**Figure 1:**
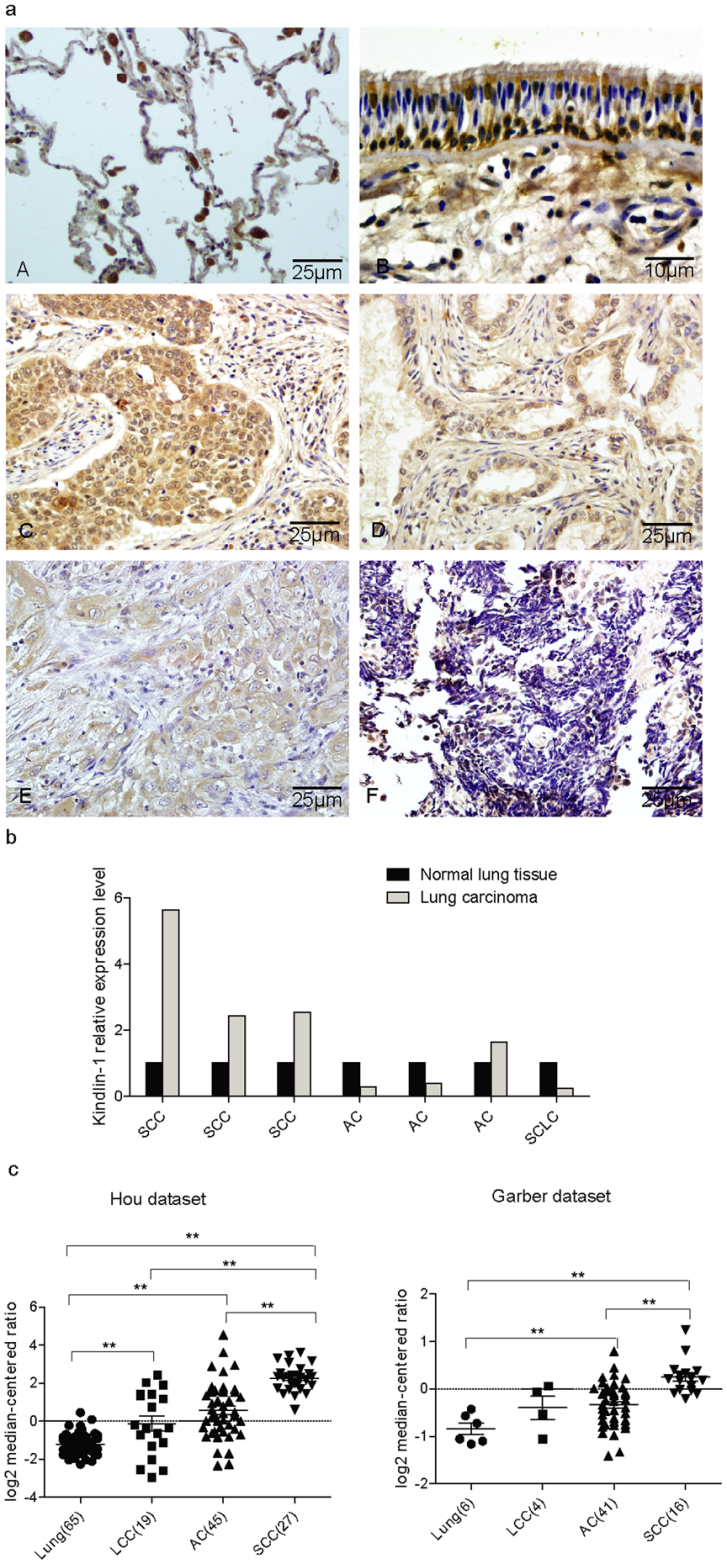
The expression pattern of Kindlin-1 in different subtypes of lung cancer. a. Kindlin-1 expression in tissues from normal and different subtypes of lung cancer examined by IHC. Photos displayed are normal lung (A), Pseudo-stratified ciliated columnar epithelium (B), SCC (C), AC (D), LCC (E), and SCLC (F). b. Determination of the mRNA levels of Kindlin-1 in the fresh clinical specimens measured by qPCR. c. Re-analyses of Kindlin-1 expression in Oncomine database. Left: Kindlin-1 expression extracted in Hou lung dataset; Right: Kindlin-1 expression extracted in Garber lung dataset. Statistical analyses between different patient groups were examined by Student's *t* test. ** represents for *p*<0.01.

Kindlin-1 expression in lung cancer has been preliminarily suggested^9,13^. However, nothing is known in detail for Kindlin-1 expression in various subtypes of lung cancer patients. To understand the posible involement of Kindlin-1 and Kindlin-2 in lung cancer progression, we first examined the expression of Kindlin-1 in lung cancer patient tissues using immnohistochemistry (IHC) with an affinity-purified polyclonal Kindlin-1 antibody, with normal lung tissues as control. We found that Kindlin-1 was not detectable in normal pulmonary alveoli and weak signal in normal lung blood vessels was seen (Fig. 1a-A). Interestingly, Kindlin-1 was found highly expressed in the cytoplasm and nuclei of cone-shaped cells and spindle cells distributed in columnar epithelia (Fig. 1a-B). However, Kindlin-1 was found weakly expressed in the colummar cells, goblet cells and not expressed in the basement memberane. In lung tumor tissues, Kindlin-1 was found highly expressed in the cytoplasm as well as membrane of SCC (Fig. 1a-C). Furthermore, Kindlin-1 was found moderately expressed in the cytoplasm of tumorous glandular epithelia in AC (Fig. 1a-D) and weakly expressed in LCC (Fig. 1a-E). Surprisingly, no expression of Kindlin-1 at all in SCLC was observed (Fig. 1a-F) (Table 2).

**Table 2:** Kindlin-1 and Kindlin-2 expressions during lung cancer progression.

Features	N = 140	KINDLIN-1 expression				*p* value		KINDLIN-2 expression				*p* value	
		0	1+	2+	3+			0	1+	2+	3+		
Tumor origin													
Epithelial-derived: NSCLC	120(85.71%)	10(8.33%)	45(37.50%)	50(41.67%)	15(12.50%)	<0.0001		38(31.67%)	55(45.83%)	21(17.50%)	6(5.00%)	0.0197	
Neuroendocrine-derived: SCLC	20(14.29%)	13(65.00%)	6(30.00%)	1(5.00%)	0.00%			11(55.00%)	8(40.00%)	1(5.00%)	0.00%		
Tumor tissue sub types													
Squamous cell carcinoma	65(46.42%)	1(1.54%)	14(21.54%)	37(56.92%)	13(20.00%)	<0.0001	<0.0001	26(40.00%)	31(47.69%)	8(12.31%)	0.00%	0.0003	0.0001
Adenocarcinoma	43(30.71%)	6(13.95%)	23(53.49%)	12(27.91%)	2(4.65%)			10(23.26%)	23(53.49%)	8(18.60%)	2(4.65%)		
Large cell undifferentiated carcinoma	12(8.57%)	3(25.00%)	8(66.67%)	1(8.33%)	0.00%			2(16.67%)	1(8.33%)	5(41.67%)	4(33.33%)		
Small cell lung carcinoma	20(14.29%)	13(65.00%)	6(30.00%)	1(5.00%)	0.00%			11(55.00%)	8(40.00%)	1(5.00%)	0.00%		
Tumor differentiation													
Differentiated carcinoma	108(90%)	7(6.48%)	37(34.26%)	49(45.37%)	15(13.89%)	<0.0001		36(33.33%)	54(50%)	16(14.81%)	2(1.85%)	<0.0001	
Large cell undifferentiated carcinoma	12(10%)	3(25.00%)	8(66.67%)	1(8.33%)	0.00%			2(16.67%)	1(8.33%)	5(41.67%)	4(33.33%)		
Well differentiated squamous cell carcinoma	4(2.86%)	0.00%	0.00%	3(75.00%)	1(25.00%)	0.0024		1(25.00%)	2(50.00%)	1(25.00%)	0.00%	0.2852	
Moderate differentiated squamous	50(35.71%)	0.00%	7(14.00%)	32(64.00%)	11(22.00%)			20(40.00%)	23(46.00%)	7(14.00%)	0.00%		
Poorly differentiated squamous cell carcinoma	11(7.86%)	1(9.09%)	7(63.64%)	2(18.18%)	1(9.09%)			5(45.45%)	6(54.55%)	0.00%	0.00%		
Well differentiated adenocarcinoma	7(5%)	3(42.86%)	1(14.29%)	1(14.29%)	2(28.57%)	0.8532		2(28.57%)	2(28.57%)	2(28.57%)	1(14.29%)	0.1759	
Moderate differentiated adenocarcinoma	28(20%)	2(7.14%)	16(57.14%)	10(35.71%)	0.00%			7(25.00%)	18(64.29%)	2(7.14%)	1(3.57%)		
Poorly differentiated adenocarcinoma	8(5.72%)	1(12.50%)	6(75.00%)	1(12.50%)	0.00%			1(12.50%)	3(37.50%)	4(50.00%)	0.00%		

**Kruskal-Wallis test (two sided)** Both of Kindlin-1 and Kindlin-2 expression are higher in NSCLC than in SCLC (*p*<0.0001). Among NSCLC, Kindlin-1 expression is low in LCC, higher in AC, and the highest is seen in SCC (*p*<0.0001), but Kindlin-2 has reverse expression trend: the level of Kindlin-2 is low in SCC, higher in AC and the highest in LCC (*p*<0.0001). The level of Kindlin-1 expression is also correlated with the level of lung cancer differentiation: differentiated SCC and AC have higher Kindlin-1 expression than undifferentiated LCC (*p*<0.0001) and High differentiated SCC has high level of Kindlin-1 expression and poorly differentiated SCC has low level of Kindlin-1 (*p* = 0.0024). This phenomenon is not obvious in AC (*p* = 0.8523). Kindlin-2 expression has no obvious relationship with the differentiation of lung cancers (*p* = 0.1759).

Furthermore, we examined the Kindlin-1 mRNA expression even freshly prepared tumor tissues from SCC, AC and SCLC of lung cancer patients. We found that SCC showed higher levels of Kindlin-1 expression than AC, and SCLC displayed the lowest expression of Kindlin-1 among various types of lung cancer patients examined (Fig. 1b).

Encouragingly, the above Kindlin-1 expression profile as determined either by immunohistochemistry or qPCR was further supported by re-analizing Kindlin-1 mRNA expression profile in patient's datasets from Oncomine Database (www.oncomine.com). After analyzing the Hou and Garber lung cancer patient's Affymetrix datasets, we found that Kindlin-1 expression in different types of NSCLC at mRNA level was significanly increased (Fig. 1c), which is in agreement with the above IHC results. These data suggest that Kindlin-1 is indeed highly expessed in SCC by examination of different patient co-horts.

### Kindlin-1 expression is correlated with cell differentiation in SCC but not in AC

**Figure 2:**
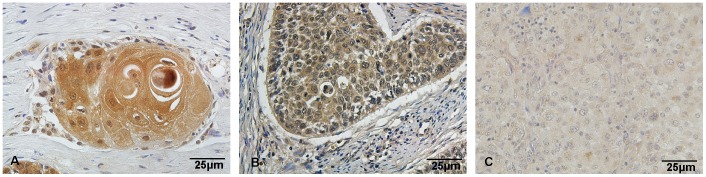
Kindlin-1 expression in different differentiation stages of squamous lung cancer. Representative photos reflecting Kindlin-1 expression in SCC have been selected and statistical analyses can be found in Table 2. A, Well differentiated SCC. B, Moderate differentiated SCC. C, Poorly differentiated SCC.

Aforementioned findings indicated that even within SCC, Kindlin-1 expression is heterogeneous. This suggests that Kindlin-1 expression may vary with the change of cell differentiation in lung cancer. To this end, we analyzed the Kindlin-1 expression in SCC with different stages of cell differentiation. Interestingly, we found that Kindlin-1 is highly expressed in the well differentiated SCC than the moderate differentiated SCC (Fig. 2-A and -B); and the moderate differentiated SCC expressed higher Kindlin-1 than the poorly differentiated SCC (Fig. 2-B and -C). However, this tendency of Kindlin-1 expression found in SCC was not applicable to AC (data not shown). Taken together, these data indicate that the higher differentiation stage responds to the higher expression of Kindlin-1 in SCC (Table 2).

### Kindlin-1 and Kindlin-2 are differentially expressed in different subtypes of lung cancer

**Figure 3:**
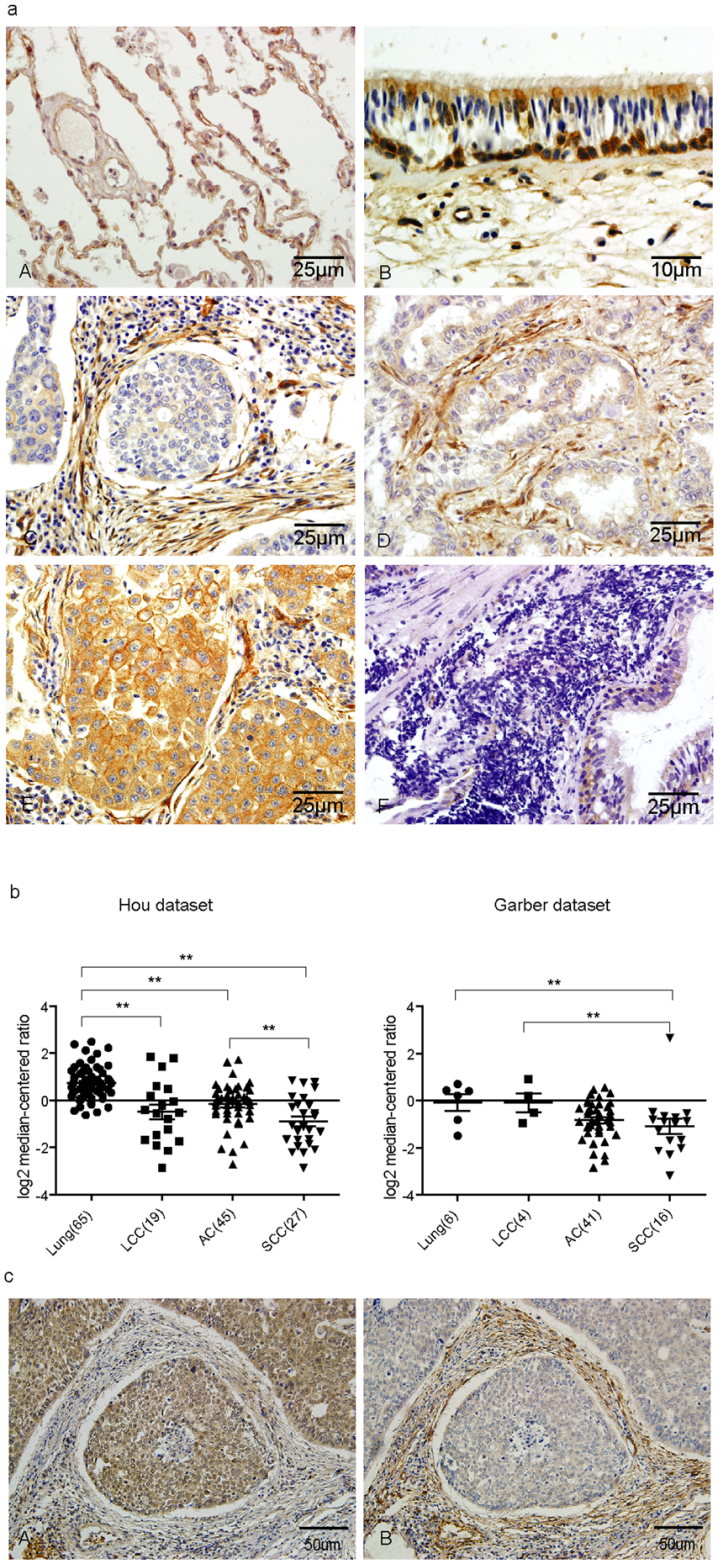
The expression pattern of Kindlin-2 in different subtypes of lung cancer. a. Kindlin-2 expression in tissues from normal and lung cancer tissues examined by IHC. Photos displayed are normal lung (A), Pseudo-stratified ciliated columnar epithelium (B), SCC (C), AC (D), LCC (E), and SCLC (F). b. Re-analyses of Kindlin-2 expression in Oncomine database. Left: Kindlin-2 expression extracted from Hou lung dataset; Right: Kindlin-2 expression extracted from Garber lung dataset. Statistical analyses between different patient groups were examined by Student's *t* test. ** represents for *p*<0.01. c. Comparison of the expressions of Kindlin-1 and Kindlin-2 in SCC from the same patient. As displayed Kindlin-2 is highly expressed in tumor stroma, but not in lung cancer cells.

Until now there is no report on Kindlin-2 expression in lung cancer cell lines as well as in lung cancer patients. It is of interest to know if both Kindlin-1 and Kindlin-2 are expressed in lung cancer patients. To this end, we examined the Kindlin-2 expression in the same panel of patient specimens aforementioned. While Kindlin-2 was found expressed in the blood vessels of the normal lung tissues (Fig. 3a-A), Kindlin-2 was found highly expressed in the cytoplasm and nuclei of cone-shaped cells (Fig. 3a-B) similar to the Kindlin-1 staining as shown in Fig. 1a-B. This indicates that both Kindlin-1 and Kindlin-2 are expressed in the normal bronchia epithilium. Interestingly, Kindlin-2 expression pattern is quite different from that of Kindlin-1 in SCC. Instead of expressed in SCC and AC, Kindlin-2 was found hihgly expressed in the tumor stroma (Fig. 3a-C and -D), which contrast sharply to the expression pattern of Kindlin-1 in these types of lung cancer. Furthermore, Kindlin-2 was found highly expressed in LCC at the cell membrane and the surrounding tumor stroma compared to the weak expression of Kindlin-1 in LCC (Fig. 3a-E). In addition, low or no positive staining of Kindlin-2 was observed in SCLC (Fig. 3a-F), which is similar to the Kindlin-1 staining in the same cell type (Fig. 1a-F). These IHC results were further strengthened by the re-analyses of Kindlin-2 mRNA expression in Oncomine databases Hou lung and Garber lung (Fig. 3b). In the series of SCC patient's tissue sections, Kindlin-1 was strongly expressed in the tumor cells, while Kindlin-2 was found expressed mainly in the tumor stroma (Fig. 3c-A, -B). These observations indicate that Kindlin-1 and Kindlin-2 are differentially expressed in lung cancer cells.

Table 2 summerized the expression of Kindlin-1 and Kindlin-2 in the tumors of various lung cancer patients. Kindlin-1 expression was positive in 84% of lung cancer patients. For the various tumor subtypes, both Kindlin-1 and Kindlin-2 showed high positive rate in NSCLC than in SCLC (*p*<0.0001). Interestingly, within NSCLC Kindlin-1 expressed at low levels in LCC, moderate in AC, and the highest expression of Kindlin-1was found in SCC (*p*<0.0001). However, Kindlin-2 has a reciprocal expression profile: the expression level of Kindlin-2 is low in SCC, moderate in AC and the highest expression of Kindlin-2 was observed in LCC (*p*<0.0001). Intriguingly, the Kindlin-1 expression was found correlated with the differentiation of tumors. SCC, composed of squamous epithelia, and AC composed of glandular epithelia were exhibited higher Kindlin-1 expression than LCC that is classified as undifferentiated carcinoma (*p*<0.0001). Furthermore, well differentiated SCC has a higher Kindlin-1 expression than poorly differentiated SCC (*p* = 0.0024). In comparison, no correlation was identified for Kindlin-1 expression with differentiation for AC (*p* = 0.8523). Similarly, Kindlin-2 expression was not found to be correlated with the differentiation of different subtypes of lung cancer (*p* = 0.1759).

### Kindlin-1 and Kindlin-2 oppositely regulate lung cancer cell progression *in vitro*

**Figure 4:**
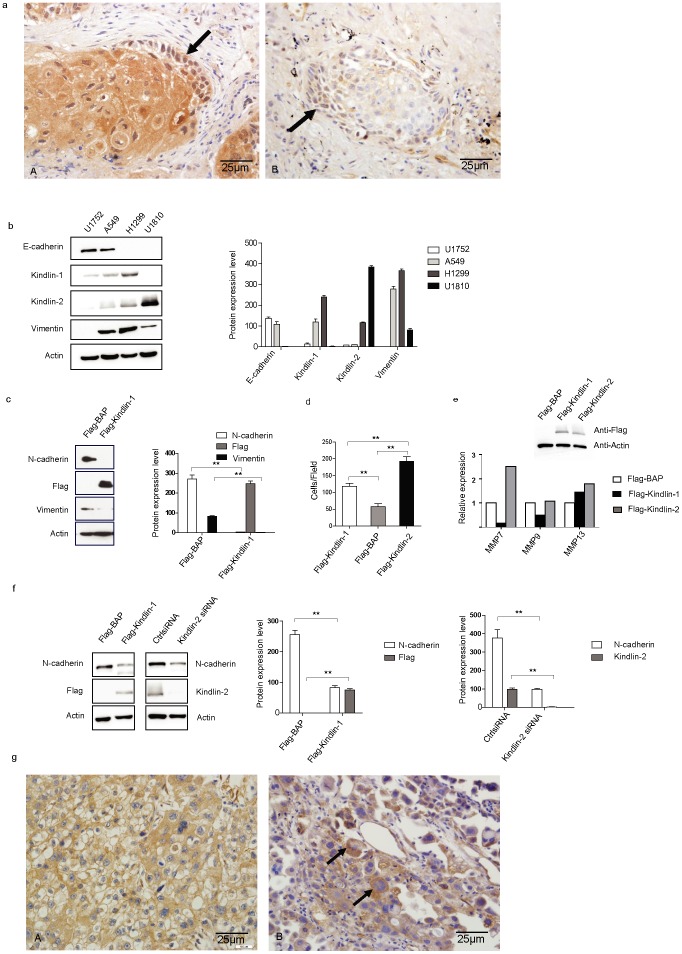
Kindlin-1 and Kindlin-2 oppositely regulate lung cancer cell migration and invasion. a. Expression of Kindlin-1 and Kindlin-2 in the secondary *de novo* SCC from an invasive SCC patient. A. Kindlin-1 expression in the *de novo* SCC. B. Kindlin-2 expression in the *de novo* SCC from the same patient as in A. b. Left panel: Differential expression of Kindlin-1, Kindlin-2, E-cadherin and Vimentin in various lung cancer cell lines controlled by actin for loading. Right panel: Quantification of the relative protein expression levels. c. Left panel: Stable expression of Kindlin-1 in H1299 cells. Indicated are mixed stable clones that express Flag-Kindlin-1. Right panel: Quantification of the relative protein levels for Kindlin-1 regulated N-cadherin and Vimentin. d. Kindlin-1 and Kindlin-2 differentially regulate migration in H1299 cells. Displayed is the quantification and plot as mean ± SEM from three independent experiments. Statistical analyses between different cell groups were examined by Student's *t* test. ** represents for *p*<0.01. e. Kindlin-1 and Kindlin-2 differentially regulate the mRNA levels of MMPs analyzed by qPCR in H1299 cells stably expressing Kindlin-1 and Kindlin-2. Insert shows expression of Kindlin-1 and Kindlin-2 in the stable mixed clones applied in qPCR. f. Left panels: Expression of Kindlin-1 downregulates N-cadherin and Knockdown of Kindlin-2 downregulates N-cadherin. Right panels: Quantification of the relative protein levels for Kindlin-1- and Kindlin-2-regulated N-cadherin. Statistical analyses between different cell groups were examined by Student's *t* test. ** represents for *p*<0.01. g. Expression of Kindlin-2 in lymph node metastasized large cell lung cancer patient. A. Primary large cell lung cancer; B. Lymph node metastasized large cell lung cancer from the same patient.

Given that Kindlin-1 and Kindlin-2 are differentially expressed in lung cancer cells, it is tempting to understand whether Kindlin-1 and Kindlin-2 also function differentially to regulate lung cancer cell progression. We previously found that Kindlin-2 was highly expressed at tumor invasive front in malignant mesothelioma [10]. To this end, we examined the expression of Kindlin-1 and Kindlin-2 in the *de novo* SCC that are localized at the secondary sites from the intra-lung dissemination of a SCC patient. As shown in Fig. 4a-A, Kindlin-1 was strongly expressed at the tumor mass of *de novo* SCC but weakly expressed at the tumor invasive front. However, Kindlin-1 staining is concentrated in the nuclei of cells at tumor invasive front of the *de novo* SCC, suggesting a cytoplasma to nucleus transition may exist for Kindlin-1 (Fig. 4a-A.Arrowed). In contrast, Kindlin-2 was not found expressed in the similar type of structure from the same patient (Fig. 4a.B). Interestingly, a cytoplasma to nucleus transition of Kindlin-2 was also found at the tumor invasive front (Fig. 4a-B.Arrowed). Collectively, these data suggest that Kindlin-1 but not Kindlin-2 is involved in the regulation of lung cancer cell colonization in SCC. Therefore, we continued to characterize the differential role of Kindlin-1 and Kindlin-2 in the regulation of lung cancer progression. We first detected the expressions of Kindlin-1 and Kindlin-2 as well as epithelial to mesenchymal transition (EMT) markers E-cadherin and Vimentin in lung cancer cell lines derived from SCC, AC and LCC and found that Kindlin-1 and Kindlin-2 were differentially expressed in lung cancer cell lines (Fig. 4b). We then established H1299 cells that stably overexpressed Kindlin-1. We found that EMT markers N-cadherin and Vimentin were downregulated in lung cancer H1299 cells stably overexpressing Kindlin-1 compared with the control cells expressing Flag-BAP (Fig. 4c), suggesting a role of Kindlin-1 in inhibition of EMT program in lung adenocarcinoma cells. Furthermore, to scrutinize the differential role of Kindlin-1 and Kindlin-2 in the regulation of lung cancer cell motility, we performed the hepatotactic migration assay on type I collagen that mediates β1 integrin-related cell migration. We found that Kindlin-1 inhibited while Kindlin-2 promoted H1299 cell migration in a Transwell assay (Fig. 4d). Meanwhile the distinct role of Kindlin-1 and Kindlin-2 in the regulation of lung cancer cell invasion was examined by determination of the expression of metalloprotenases including MMP7, MMP9 and MMP13. MMP7 and MMP9 were found to be downregulated with the ectopic expression of Kindlin-1, whereas MMP7 and MMP13 were upregulated with the ectopic expression of Kindlin-2 (Fig. 4e). In a transient transfection, overexpression of Kindlin-1 led to downregulation of N-cadherin (Fig. 4f left panel), suggesting an inhibitory role of Kindlin-1 on EMT occurrence. However, knockdown of endogenous Kindlin-2 decreased the level N-cadherin (Fig. 4f left panel), an effect that is equivalent to the overexpression of Kindlin-1. Furthermore, we examined the expression levels of Kindlin-2 in primary and secondary LCC and found that Kindlin-2 was strongly expressed in lymph nodes of metastasized LCC (Fig. 4g-B, arrowed) compared to the primary tumor from the same patient (Fig. 4g-A). This finding suggested that high level of Kindlin-2 expression may correspond to a high potential of LCC invasion. Taken together, these data indicated that the differential expressions of Kindlin-1 and Kindlin-2 in lung cancer patients correspond to an opposite regulation on cell migration as well as on the cell invasive capability.

### Kindlin-1 and Kindlin-2 oppositely regulate lung cancer cell growth in an *in vivo* mouse xenograft model

**Figure 5:**
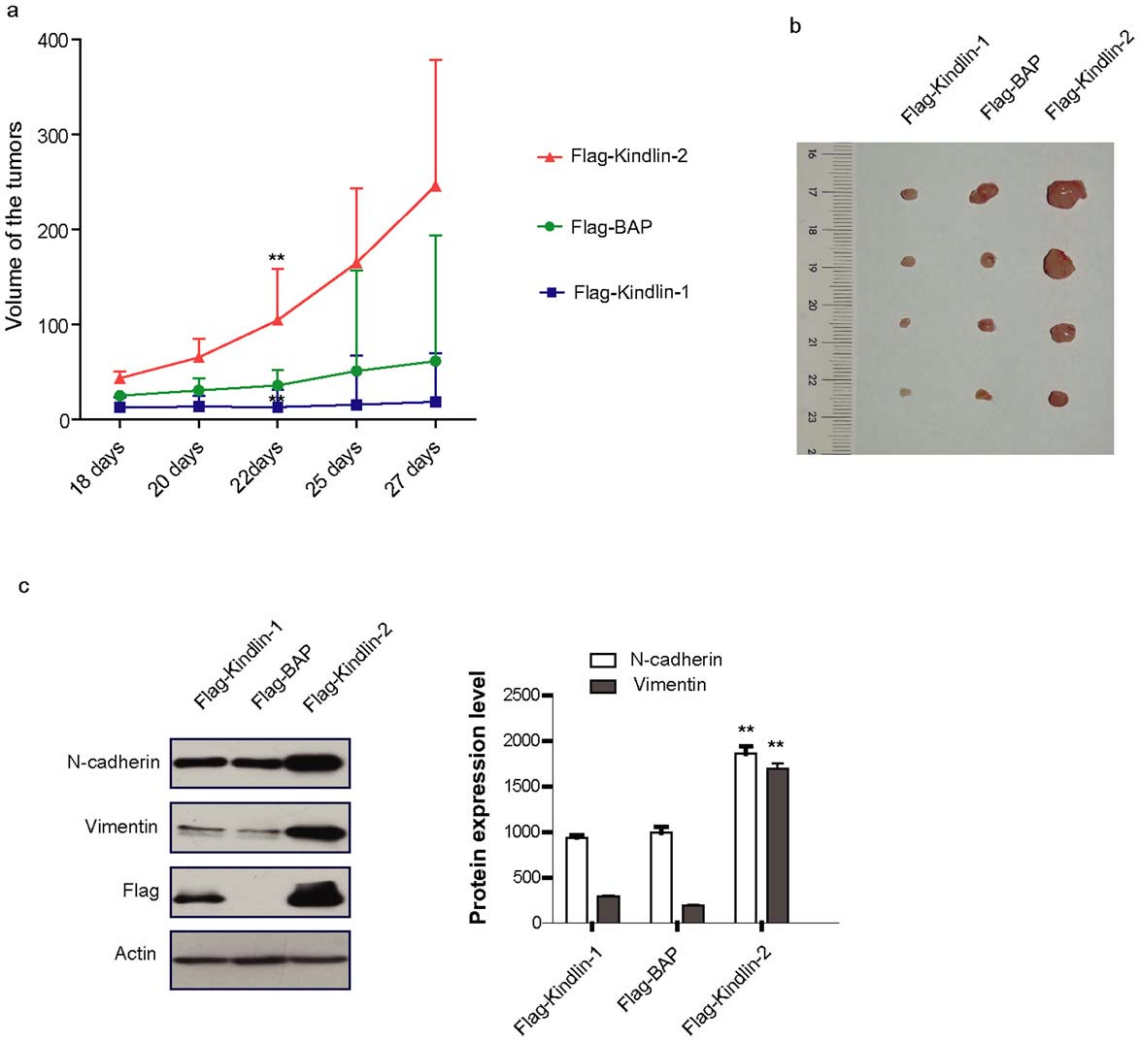
Kindlin-1 and Kindlin-2 oppositely regulate tumor growth in implanted xenograft. a. Tumor growth curve. The tumor volume of H1299-Flag-Kindlin-1 group is significantly smaller than the Flag-BAP group (*p*<0.01 as analyzed by Student's *t* test) at day 22 and meanwhile the volume of Flag-Kindlin-2 group is obviously larger than the Flag-BAP group (*p*<0.01 as analyzed by Student's *t* test). b. Tumors recovered from mice at day 27. c. Left panel: Determination of expressions of N-cadherin and Vimentin from tumor xenografts stably expressing Kindlin-1, Kindlin-2 and the control BAP by Western blot analyses. Right panel: Quantification of the relative protein levels for Kindlin-1- and Kindlin-2-regulated N-cadherin and Vimentin. Statistical analyses between different tumor groups were examined by Student's *t* test. ** represents for *p*<0.01.

Based on the above finding that Kindlin-1 and Kindlin-2 were differentially expressed in lung cancer patient specimens, and oppositely regulated lung cancer cell migration *in vitro*, we were eager to know whether Kindlin-1 and Kindlin-2 play a distinct role in the regulation of tumor growth *in vivo*. To this end, the mouse tumor xenografts with Kindlin-1 overexpression grew slower and the sizes of tumors were smaller than that of the control (Fig. 5a and b). However, tumors with Kindlin-2 overexpression grew much faster and the sizes of tumors were much larger than that of the control (Fig. 5a and b). Interestingly, tumor tissues recovered from the mouse xenografts also displayed increased N-cadherin and Vimentin expression in the Kindlin-2 overexpressing tumors, with no changes for N-cadherin and Vimentin in Kindlin-1 overexpressed or the control tumors (Fig. 5c). These data clearly demonstrated that Kindlin-1 and Kindlin-2 not only oppositely regulated tumor growth but also the tumor invasive potential in the *in vivo* mouse xenograft model.

## Discussion

Kindlin-1 expression had been known to be related to lung cancer for many years [9]. However, until now there is no detailed study of Kindlin-1 expression in various types of lung cancer. In this report, we scrutinized the Kindlin-1 expression in NSCLC including SCC, AC and LCC as well as SCLC. Our findings have demonstrated that Kindlin-1expression in lung cancer is correlated with the origin of the cells. For example, Kindlin-1 is higher expressed in epithelium-originated NSCLC but not expressed or lower expressed in neuroendocrine-originated SCLC, suggesting that Kindlin-1 is mainly expressed in epithelia originated SCC and AC. In comparison, LCC is non-squamous, non-adenomatous type of lung cancer that is undifferentiated and is usually highly malignant [22]. Interestingly, in this report Kindlin-1 expression was identified higher in SCC than that of AC and LCC. In agreement with this finding, data from Oncomine cancer expression database support this conclusion (https://www.oncomine.org/resource). Re-analyses of Hou and Garber lung datasets indicated that SCC express higher Kindlin-1 than adenocarcinoma and large cell lung cancer, whereas large cell lung cancer expresses low level of Kindlin-1. Re-analyses of Kindlin-1 expression in other datasets including TCGA, Su multicancer and Zhu lung databases (data not shown) of Oncomine displayed the same tendency. Collectively, Kindlin-1 expression in SCC is higher than AC at both mRNA and protein levels. In addition, the expression of Kindlin-1 is higher in well differentiated SCC than in undifferentiated LCC. These data suggest that Kindlin-1 expression is correlated with lung cancer differentiation, and Kindlin-1 could be used as a differentiation marker for SCC. Therefore, it is possible to control the differentiation of lung squamous epithelia and the SCC through regulation of Kindlin-1 expression. For the translational relevance of Kindlin-1 expression and, well differentiated SCC has been known to have low metastatic rate and low relapse after surgery and usually sensitive to radiotherapy and chemotherapy, and predicts good prognosis [23]. However, the non-squamous, non-adenomotous undifferentiated LCC displayed early metastasis, insensitive to radiotherapy and chemotherapy and predicts poor prognosis for the patients [22].

**Figure 6:**
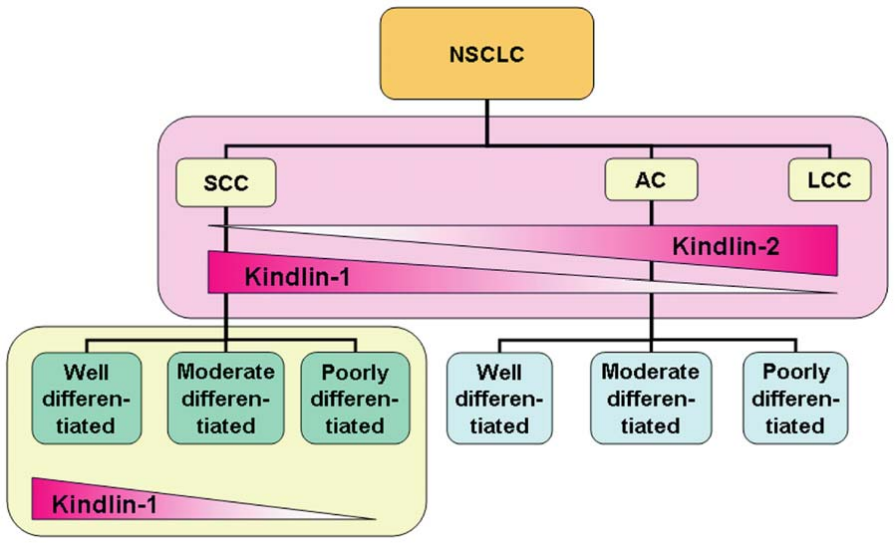
A working model for the opposite role of Kindlin-1 and Kindlin-2 in lung cancer progression. In the model, Kindlin-1 expression decreased from well differentiated to poorly differentiated SCC on one hand, and decreased as well from SCC to LCC on the other hand. In contrast, Kindlin-2 expression increased from SCC to LCC.

Interestingly, the expression pattern of Kindlin-2 in lung cancer is different from that of Kindlin-1. Instead of expression in tumor cells, Kindlin-2 is mainly expressed in the stroma of various types of lung cancer. Kindlin-2 expression was found in fibroblasts and smooth muscle cells of the blood vessels. We demonstrated that in this report Kindlin-1 and Kindlin-2 are differentially expressed in various types of lung cancer. Like Kindlin-1, Kindlin-2 is also higher expressed in NSCLC than in SCLC, indicating that Kindlin-2 tends to express in epithelia-originated but not in neuroendocrine-originated lung cancers. However, Kindlin-2 expression profile was just opposite with that of Kindlin-1 in NSCLC. Kindlin-2 expression levels obey an order of LCC>AC>SCC, suggesting that Kindlin-2 plays an opposite role in NSCLC: higher Kindlin-2 expression corresponds to a worse lung cancer phenotype. Due to the inadequate cases of LCC in our study we could not clearly establish the relationship between Kindlin-2 expression level and the outcome of LCC patients. However, the positive correlation for Kindlin-2 expression with poor disease outcome was supported by data from Oncomine database. By re-analyzing the Hou and Garber lung datasets we found that Kindlin-1 expression in SCC is high while Kindlin-2 expression is low in the same cohort of SCC patients. It is generic that Kindlin-2 expression is lower in SCC than that of AC. Taken together, these findings strongly indicated that Kindlin-1 and Kindlin-2 play an opposite role in the regulation of NSCLC behaviors. The differential expression and counteracting role of Kindlin-1 and Kindlin-2 in lung cancers were depicted in a working model as shown in Figure 6. Furthermore, it seems that the sum of Kindlin-1 and Kindlin-2 remains constant in an NSCLC patient, thus the biological role of the molecular switch between Kindlin-1 and Kindlin-2 leaves an open question for future investigations.

Intriguingly, Kindlin-1has recently been reported to promote breast cancer lung metastasis [13], a role that is just opposite for what we found in lung cancer. This is the first report that a counteracting role of Kindlin-1 in human cancers. To this end, we found that Kindlin-1 exhibits an inhibitory role for tumor growth and invasion in lung cancer. Therefore, it was of particular interest to answer why Kindlin-1 functions adversely in breast cancer as that of it in lung cancer. As a possible mechanism accounting for the diverse roles of Kindlin-1 in cancers, we identified that Kindlin-1 downregulates Wnt signaling component Axin2 and upregulates tight junction molecules such as Claudin-1 and -3 in lung cancer cells (Zhan et al., data not shown). Adversely, Kindlin-1 upregulates Wnt signaling component β-catenin and EMT markers Vimentin and fibronectin in breast cancer cells [13]. In a small cohort of lung cancer patients, Sin et al also found that Kindlin-1 is overexpressed in lung primary tumors and Kindlin-1 expression is strongly associated with metastasis-free survival of patients with lung adenocarcinomas [13]. The findings of Sin et al indicated that Kindlin-1 is overexpressed in primary tumors but not in metastatic tumors in lung cancer, which is in agreement with our conclusion that Kindlin-1 is overexpressed in well differentiated SCC but low or not expressed in large cell or small cell lung cancers.

Besides the counteracting role of Kindlin-1 in breast cancer and lung cancer cells, surprisingly we uncovered the opposite role of Kindlin-1 and Kindlin-2 in the regulation of lung cancer progression. Kindlin-1 inhibited whereas Kindlin-2 promoted lung cancer cell migration and invasion in an in vitro assay. The *in vivo* tumor implant experiment clearly demonstrated that Kindlin-1 prohibited but Kindlin-2 accelerated xenograft tumor growth. Mechanistically, Kindlin-1 downregulated epithelial to mesenchymal transition markers N-cadherin and Vimentin, whereas Kindlin-2 expression upregulated these markers as indicated in Fig. 4f. These findings suggest that Kindlin-1 and Kindlin-2 play an opposite role in the regulation of EMT process, which account for the mechanistic interpretation for Kindlin-1 and Kindlin-2 involvement in the regulation of lung cancer cell invasion. Furthermore, in clinical samples we found that Kindlin-1 expression is lower at the tumor invasive front as shown in Fig. 4a, suggesting that Kindlin-1 inhibited lung cancer cell migration, invasion as well as tumor growth at these cellular motile structures. In comparison, Kindlin-2 is highly expressed at these structures and apparently promoted lung cancer cell migration and invasion as previous reports [10], [12]. All together, at layers of cells, xenograft tumor implants and patient tumor tissue samples Kindlin-1 and Kindlin-2 displayed their opposite biological functions in lung cancer.

In summary, in this study we found that Kindlin-1 and Kindlin-2 play distinct roles in lung cancer progression. Kindlin-1 predominantly expressed in well differentiated NSCLC and inhibited the malignant progression including tumor invasion and growth, whereas Kindlin-2 mainly expressed in poorly differentiated NSCLC and promoted tumor invasion and growth. Kindlin-1 can be potentially used as a marker for evaluation of lung cancer differentiation while Kindlin-2 may be a hopeful therapeutic target for LCC.
